# Supplementary material for: Comparing driving behavior of humans and autonomous driving in a professional racing simulator
Source: PLoS One. 2021 Feb 3;16(2):e0245320. doi: 10.1371/journal.pone.0245320 (PMC7857611; doi:10.1371/journal.pone.0245320)
Supplement: S1 File — (PDF) [file pone.0245320.s001.pdf]

Driving Evaluation

Thanks for taking part in this evaluation!

* If you have any questions, please feel free to ask.
* Required

1.

____

General Information

2. How's your English? *

   You will need some basic English skills to complete the evaluation. If you don't understand some words, it's not a problem at all, just ask at any time.

   Mark only one oval.

   - ◯ Ich spreche kein Englisch.
   - ◯ Basic
   - ◯ Okay
   - ◯ Fluent
   - ◯ I'm a native speaker

3. Do you have a driving licence? *

   Mark only one oval.

   - ◯ No
   - ◯ Yes

Real car driving experience

4. How many hours a week do you drive a real car? *

____

5. Number of years of experience with normal driving *

____

6. Annual kilometers

____

7. Number of accidents (optional)

____

8. Number of traffic fines (optional)

____

9. Rate your driving skills in real life?

Mark only one oval.

	1	2	3	4	5	6	7	8	9	10	
Very Poor	⬭	⬭	⬭	⬭	⬭	⬭	⬭	⬭	⬭	⬭	Excellent

Real racing experience

10. Number of years of experience with racing cars

____

11. Number of participated go-kart (karting) races

____

Driving simulators and games

12. How many hours a week do drive in a racing simulator? *

____

13. How many hours a week do play racing games? *

____

14. If you play driving racing video games, what controllers do you use?

Check all that apply.

- [ ] Gamepad
- [ ] Steering wheel
- [ ] Driving pedals
- [ ] keyboard/mouse

15. Rate your driving skills in video racing games?

Mark only one oval.

	1	2	3	4	5	6	7	8	9	10	
Very Poor	◯	◯	◯	◯	◯	◯	◯	◯	◯	◯	Excellent

16. Rate your driving skills in a simulator?

Mark only one oval.

	1	2	3	4	5	6	7	8	9	10	
Very Poor	◯	◯	◯	◯	◯	◯	◯	◯	◯	◯	Excellent

This content is neither created nor endorsed by Google.

Google Forms
