# Supplementary material for: Comparing driving behavior of humans and autonomous driving in a professional racing simulator
Source: PLoS One. 2021 Feb 3;16(2):e0245320. doi: 10.1371/journal.pone.0245320 (PMC7857611; doi:10.1371/journal.pone.0245320)
Supplement: S2 File — (PDF) [file pone.0245320.s002.pdf]

NASA TLX

Thanks for taking part in this evaluation!

* If you have any questions, please feel free to ask.

* Required

1\.

How difficult was driving the car (Session 1)?

The following assessment is used to measure your personal opinion on how much workload was required of you during the task you just completed. There is no right or wrong answer.

2\. Mental Demand *

How mentally demanding was the task?

Mark only one oval.

	0	1	2	3	4	5	6	7	8	9	10	
Very Low	⬭	⬭	⬭	⬭	⬭	⬭	⬭	⬭	⬭	⬭	⬭	Very High

3\. Physical Demand *

How physically demanding was the task?

Mark only one oval.

	0	1	2	3	4	5	6	7	8	9	10	
Very Low	⬭	⬭	⬭	⬭	⬭	⬭	⬭	⬭	⬭	⬭	⬭	Very High

4\. Temporal Demand *

How hurried or rushed was the pace of the task?

Mark only one oval.

	0	1	2	3	4	5	6	7	8	9	10	
Very Low	⬭	⬭	⬭	⬭	⬭	⬭	⬭	⬭	⬭	⬭	⬭	Very High

5. Performance *

How successful were you in accomplishing what you were asked to do?

Mark only one oval.

	0	1	2	3	4	5	6	7	8	9	10	
Failure	◯	◯	◯	◯	◯	◯	◯	◯	◯	◯	◯	Perfect

6. Effort *

How hard did you have to work to accomplish your level of performance?

Mark only one oval.

	0	1	2	3	4	5	6	7	8	9	10	
Very Low	◯	◯	◯	◯	◯	◯	◯	◯	◯	◯	◯	Very High

7. Frustration *

How insecure, discouraged, irritated, stressed, and annoyed were you?

Mark only one oval.

	0	1	2	3	4	5	6	7	8	9	10	
Very Low	◯	◯	◯	◯	◯	◯	◯	◯	◯	◯	◯	Very High

8. Any comments? What was good / bad / unexpected / difficult?

How difficult was driving the car (Session 2)?

The following assessment is used to measure your personal opinion on how much workload was required of you during the task you just completed. There is no right or wrong answer.

9. Mental Demand *

How mentally demanding was the task?

Mark only one oval.

	0	1	2	3	4	5	6	7	8	9	10	
Very Low												Very High

10. Physical Demand *

How physically demanding was the task?

Mark only one oval.

	0	1	2	3	4	5	6	7	8	9	10	
Very Low												Very High

11. Temporal Demand *

How hurried or rushed was the pace of the task?

Mark only one oval.

	0	1	2	3	4	5	6	7	8	9	10	
Very Low												Very High

12. Performance *

How successful were you in accomplishing what you were asked to do?

Mark only one oval.

	0	1	2	3	4	5	6	7	8	9	10	
Failure												Perfect

13. Effort *

How hard did you have to work to accomplish your level of performance?

Mark only one oval.

	0	1	2	3	4	5	6	7	8	9	10	
Very Low	◯	◯	◯	◯	◯	◯	◯	◯	◯	◯	◯	Very High

14. Frustration *

How insecure, discouraged, irritated, stressed, and annoyed were you?

Mark only one oval.

	0	1	2	3	4	5	6	7	8	9	10	
Very Low	◯	◯	◯	◯	◯	◯	◯	◯	◯	◯	◯	Very High

15. Any comments? What was good / bad / unexpected / difficult?

____

____

____

____

____

How difficult was driving the car (Session 3)?

The following assessment is used to measure your personal opinion on how much workload was required of you during the task you just completed. There is no right or wrong answer.

16. Mental Demand *

How mentally demanding was the task?

Mark only one oval.

	0	1	2	3	4	5	6	7	8	9	10	
Very Low	◯	◯	◯	◯	◯	◯	◯	◯	◯	◯	◯	Very High

17. Physical Demand *

How physically demanding was the task?

Mark only one oval.

	0	1	2	3	4	5	6	7	8	9	10	
Very Low												Very High

18. Temporal Demand *

How hurried or rushed was the pace of the task?

Mark only one oval.

	0	1	2	3	4	5	6	7	8	9	10	
Very Low												Very High

19. Performance *

How successful were you in accomplishing what you were asked to do?

Mark only one oval.

	0	1	2	3	4	5	6	7	8	9	10	
Failure												Perfect

20. Effort *

How hard did you have to work to accomplish your level of performance?

Mark only one oval.

	0	1	2	3	4	5	6	7	8	9	10	
Very Low												Very High

21. Frustration *

How insecure, discouraged, irritated, stressed, and annoyed were you?

Mark only one oval.

	0	1	2	3	4	5	6	7	8	9	10	
Very Low												Very High

22. Any comments? What was good / bad / unexpected / difficult?

How difficult was driving the car (Session 4)?

The following assessment is used to measure your personal opinion on how much workload was required of you during the task you just completed. There is no right or wrong answer.

23. Mental Demand *

How mentally demanding was the task?

Mark only one oval.

	0	1	2	3	4	5	6	7	8	9	10	
Very Low												Very High

24. Physical Demand *

How physically demanding was the task?

Mark only one oval.

	0	1	2	3	4	5	6	7	8	9	10	
Very Low												Very High

25. Temporal Demand *

How hurried or rushed was the pace of the task?

Mark only one oval.

	0	1	2	3	4	5	6	7	8	9	10	
Very Low												Very High

26. Performance *

How successful were you in accomplishing what you were asked to do?

Mark only one oval.

	0	1	2	3	4	5	6	7	8	9	10	
Failure												Perfect

27. Effort *

How hard did you have to work to accomplish your level of performance?

Mark only one oval.

	0	1	2	3	4	5	6	7	8	9	10	
Very Low												Very High

28. Frustration *

How insecure, discouraged, irritated, stressed, and annoyed were you?

Mark only one oval.

	0	1	2	3	4	5	6	7	8	9	10	
Very Low												Very High

29. Any comments? What was good / bad / unexpected / difficult?

____

____

____

____

____

How difficult was driving the car (Session 5)?

The following assessment is used to measure your personal opinion on how much workload was required of you during the task you just completed. There is no right or wrong answer.

30. Mental Demand *

How mentally demanding was the task?

Mark only one oval.

	0	1	2	3	4	5	6	7	8	9	10	
Very Low	◯	◯	◯	◯	◯	◯	◯	◯	◯	◯	◯	Very High

31. Physical Demand *

How physically demanding was the task?

Mark only one oval.

	0	1	2	3	4	5	6	7	8	9	10	
Very Low	◯	◯	◯	◯	◯	◯	◯	◯	◯	◯	◯	Very High

32. Temporal Demand *

How hurried or rushed was the pace of the task?

Mark only one oval.

	0	1	2	3	4	5	6	7	8	9	10	
Very Low	◯	◯	◯	◯	◯	◯	◯	◯	◯	◯	◯	Very High

33. Performance *

How successful were you in accomplishing what you were asked to do?

Mark only one oval.

	0	1	2	3	4	5	6	7	8	9	10	
Failure	◯	◯	◯	◯	◯	◯	◯	◯	◯	◯	◯	Perfect

34. Effort *

How hard did you have to work to accomplish your level of performance?

Mark only one oval.

	0	1	2	3	4	5	6	7	8	9	10	
Very Low	◯	◯	◯	◯	◯	◯	◯	◯	◯	◯	◯	Very High

35. Frustration *

How insecure, discouraged, irritated, stressed, and annoyed were you?

Mark only one oval.

	0	1	2	3	4	5	6	7	8	9	10	
Very Low	◯	◯	◯	◯	◯	◯	◯	◯	◯	◯	◯	Very High

36. Any comments? What was good / bad / unexpected / difficult?

____

____

____

____

____

This content is neither created nor endorsed by Google.

Google Forms
