# Supplementary material for: Comparing driving behavior of humans and autonomous driving in a professional racing simulator
Source: PLoS One. 2021 Feb 3;16(2):e0245320. doi: 10.1371/journal.pone.0245320 (PMC7857611; doi:10.1371/journal.pone.0245320)
Supplement: Attachment — Submitted filename: Response to Reviewers.pdf [file pone.0245320.s003.pdf]

Rebuttal Letter

The authors would like to thank the reviewers for their fair reviews and detailed list of constructive suggestions. We greatly appreciate the opportunity to submit a major revision of our paper for your renewed consideration. Below we respond to reviewers' comments and point to the corresponding changes we made in the paper.

Reviewer'1 (R1) concerns:

1. Authors need justify the sample size selection, i.e., 14 participants.

Our response: As we wanted to have users with various driving skills (including experts) and we wanted to cluster them and analyse their behaviour based on their performance, it was important that the number of users with various expertise was not extremely skewed. Unfortunately, expert/professional racing drivers are difficult to recruit and we were able to recruit 2 of them. Given that we did not want to have an extremely skewed number of participants in each experience group, we did not recruit a lot of amateur drivers. We extended the description of section **Participants (see page 12)** to provide our argumentation about the number of participants.

2. What's the motivation for autonomous driving for race cars?

Our response: We realise that in our first submission we did not emphasise the importance of autonomous race cars even though we think it is very relevant. We thank the reviewer for pointing this out. We extended the Introduction with a full paragraph (paragraph 3) which elaborates our motivation as well as the relevance of autonomous racing cars.

3. Authors have briefly reviewed the state of the art on race cars. But I do suggest to add one section to review the general state of the art in regular car driving simulator research and this will be beneficial to researchers.

Our response: We thank the reviewer for the suggestion and we fully agree that extending the related work in the suggested areas is very beneficial for the reader. We considerably extended our related work with three more sections which cover the related work in autonomous driving, methods comparing human and machines as well as simulators.

4. The autonomous driving part is difficult to follow. Authors may be reorganize that session to ensure it is easy to follow.
Our response: We rewrote the major part of the autonomous driving part the in related work with the aim of improving the readability of this section. We also splitted this part into related work and background and moved the background to the "Autonomous driving" section

5. In all figures, I suggest to add units for horizontal and vertical axes.
Our response: We added the units for the horizontal and vertical axes in all figures as the reviewer suggested.

6. Was the autonomous driving, reinforcement machine learning session, trained by the results of human drivers (participants)? If so what's the meaning of comparing their driving behaviors?

Our response: apart from a hint of the racing lin**e,** the autonomous driving (reinforcement learning) did not use the data from the human drivers but learned on its own. There is only one analysis where we use the

model trained by human data that predicts the lap time and use the data from self-driving agents to see how accurate the prediction would be. This small trial investigated whether the model learned on humans can be used by RL agents to predict the lap time which turns out not to be the case. The point of this small trial was to investigate whether their behaviours of humans and autonomous agents are similar and the conclusion of this trial was that the driving behaviours are very different. This trial is very consistent also with the results in Table 4 where it is obvious that the features that predict the performance of a lap are very different when comparing RF agents with human drivers.

Reviewer'2 (R1) concerns:

1. As far as I see, the authors do not completely answer the question they proposed in the contribution part, or make readers confused. For example, the question like "what can we learn from humans to boost the machine performance" need to further discussed as currently only limited conclusion can be made.

Our response: We understand the reviewer's concern and we appreciate pointing it out. Thus, we added a full section (Lessons Learned) elaborating the importance of our findings and how the knowledge produced is useful in self-driving, racing, training etc… In addition, we changed our claim in the abstract about "boosting the machine performance" with a more appropriate description which better matches the contribution of our work: "*Our study investigates the human driving patterns with the goal of finding traces that could help improve the performance of reinforcement learning approaches*".

2. The literature review part show the authors gain a good knowledge in the motorsports and racing, and the RL algorithms, etc. However, one important part is missing, which are review of existing studies on the analysis between human and machine, particular some important conclusions, connections, and methodologies.

Our response: We thank the reviewer for the suggestion and as suggested, we extended our related work with a full section on humans vs machines which included the studies we could find that compare those two entities. Please note that the studies in this direction are quite rare.

3. More discussions and data visualization are expected for the comparison of the human performance and machine performance. For example, for the Barcelona-Catalunya circuit, is there any moment that human and automation show similar driving performance or characteristics, and when will the performance be significant different with each other?

Our response: We thank the reviewer for the suggestion and we realise that more analysis would improve the quality of our paper. Therefore, we added six more figures (Figures 8, 9, 10, 11, 12, 13, 17 and 18) comparing the performance of good vs bad drivers among RF agents as well as human drivers. In addition, we added a full section (High vs Poor Performance Driving Patterns) discussing those figures and relating the discussion with the features in Table 4. The newly added figures are very consistent with the features displayed in Table 4, which makes our finding even more reliable and conclusive.

4. The authors are encourage to summarize the most important knowledge that human driving can be used to improve the machine training, and discuss any application limitation.

Our response: We thank the reviewer for the suggestion. In the revised version, we added a full section (Lessons Learned) elaborating the importance of our findings and how the knowledge produced is useful in self-driving, racing, training etc

5. The manuscript need to be carefully checked in terms of English writing and citation. For example, in page 20, line 618, which table is refer to?

Our response: We understand the concern and apologise for the inconvenience. Although the authors worked diligently and hard on this paper, there were still errors as none of the authors is a native English speaker. Nevertheless, we understand the importance of providing content with a good readability aspect of it as it may negatively impact the quality of the paper. Thus, in addition to correcting them, we used an external person to full proof-read the manuscript.

General Issues and concerns:

1. Please ensure that your manuscript meets PLOS ONE's style requirements, including those for file naming.

Our response: We used the latest official template of PLOS ONE

2. We note that you have stated that you will provide repository information for your data at acceptance. Should your manuscript be accepted for publication, we will hold it until you provide the relevant accession numbers or DOIs necessary to access your data. If you wish to make changes to your Data Availability statement, please describe these changes in your cover letter and we will update your Data Availability statement to reflect the information you provide.

Our response: We maintain our claim regarding making our data publicly available as we strongly believe in the importance of publicly available data due to the reproducibility of our experiment and reusability of data for other purposes. We will provide the DOIs upon acceptance.

3. Thank you for stating the following in the Competing Interests/Financial Disclosure section* (delete as necessary):

"The funding for this work was awarded to Adrian Remonda. This research was partially funded by AVL GmbH (www.avl.com) and Know-Center GmbH (www.know-center.at). Know-Center is funded within the Austrian COMET Program - Competence Centers for Excellent Technologies - under the auspices of the Austrian Federal Ministry of Transport, Innovation and Technology, the Austrian Federal Ministry of Economy, Family and Youth and by the State of Styria. COMET is managed by the Austrian Research Promotion Agency FFG (https://www.ffg.at/en). The sponsors and funders played no role in the study design, data collection and analysis, decision to publish, or preparation of the manuscript."

We note that one or more of the authors have an affiliation to the commercial funders of this research study : "Know-Center GmbH"

a) Please provide an amended Funding Statement declaring this commercial affiliation, as well as a statement regarding the Role of Funders in your study. If the funding organization did not play a role in the study design, data collection and analysis, decision to publish, or preparation of the manuscript and only provided financial support in the form of authors' salaries and/or research materials, please review your statements relating to the author contributions, and ensure you have specifically and accurately indicated the role(s) that these authors had in your study. You can update author roles in the Author Contributions section of the online submission form.

Please also include the following statement within your amended Funding Statement.

“The funder provided support in the form of salaries for authors [insert relevant initials], but did not have any additional role in the study design, data collection and analysis, decision to publish, or preparation of the manuscript. The specific roles of these authors are articulated in the ‘author contributions’ section.”

If your commercial affiliation did play a role in your study, please state and explain this role within your updated Funding Statement.

Our response:

Financial Disclosure: The funding for this work was awarded to Adrian Remonda. This research was partially funded by AVL GmbH (www.avl.com) and Know-Center GmbH (www.know-center.at).Know-Center is funded within the Austrian COMET Program - Competence Centers for ExcellentTechnologies - under the auspices of the Austrian Federal Ministry of Transport, Innovation andTechnology, the Austrian Federal Ministry of Economy, Family and Youth and by the State of Styria.COMET is managed by the Austrian Research Promotion Agency FFG (https://www.ffg.at/en). The Sponsors and funders played no role in the study design, data collection and analysis, decision to publish, or preparation of the manuscript. The funder provided support in the form of salaries for authors Adrian Remonda, but did not have any additional role in the study design, data collection and analysis, decision to publish, or preparation of the manuscript. The specific roles of these authors are articulated as follows. Each named author (Adrian Remonda, Eduardo Veas and Granit Luzhnica) has substantially contributed to conducting the underlying research and drafting this manuscript. In Detail, the three authors contributed to the research methodology and experimental plan. AdrianRemonda implemented the algorithms and executed the study. The three authors contributed to data analysis and reporting. Additionally, the named authors have no conflict of interest, financial or otherwise.

b) Please also provide an updated Competing Interests Statement declaring this commercial affiliation and your additional commercial funder (AVL GmbH) along with any other relevant declarations relating to employment, consultancy, patents, products in development, or marketed products, etc.

Within your Competing Interests Statement, please confirm that this commercial affiliation does not alter your adherence to all PLOS ONE policies on sharing data and materials by including the following statement: "This does not alter our adherence to PLOS ONE policies on sharing data and materials.” (as detailed online in our guide for authors http://journals.plos.org/plosone/s/competing-interests). If this adherence statement is not accurate and there are restrictions on sharing of data and/or materials, please state these. Please note that we cannot proceed with consideration of your article until this information has been declared.

Please include both an updated Funding Statement and Competing Interests Statement in your cover letter. We will change the online submission form on your behalf.

Please know it is PLOS ONE policy for corresponding authors to declare, on behalf of all authors, all potential competing interests for the purposes of transparency. PLOS defines a competing interest as anything that interferes with, or could reasonably be perceived as interfering with, the full and objective presentation, peer review, editorial decision-making, or publication of research or non-research articles submitted to one of the journals. Competing interests can be financial or non-financial, professional, or personal. Competing interests can arise in relationship to an organization or another person. Please follow this link to our website for more details on competing interests:
http://journals.plos.org/plosone/s/competing-interests

Our response:

Competing Interests: the authors declare having a commercial affiliation with AVL GmbH and Know-Center GmbH and declare no having competing interests. The authors also declare that his affiliation does not alter the adherence to PLOS ONE policies on sharing data and materials

4. Please include captions for your Supporting Information files at the end of your manuscript, and update any in-text citations to match accordingly. Please see our Supporting Information guidelines for more information: http://journals.plos.org/plosone/s/supporting-information.

Our response: Thank you for the note. In this version, we included the captions of Supporting Information according to the recommendation.
