# Supplementary material for: Comparing driving behavior of humans and autonomous driving in a professional racing simulator
Source: PLoS One. 2021 Feb 3;16(2):e0245320. doi: 10.1371/journal.pone.0245320 (PMC7857611; doi:10.1371/journal.pone.0245320)
Supplement: Attachment — Submitted filename: Response to Reviewers.pdf [file pone.0245320.s004.pdf]

Rebuttal Letter

The authors would like to thank the reviewers for their fair reviews and detailed list of constructive suggestions. We greatly appreciate the opportunity to submit a major revision of our paper for your renewed consideration. Below we respond to reviewers' comments and point to the corresponding changes we made in the paper. The line numbers below refer to the line numbers in the manuscript with track changes.

Reviewer concerns:

1. *In line (18) and line (35), the authors tried to discuss about passenger self-driving cars as they are less risky to magnify that the racing cars are comparatively in their maximum risk ceiling. But the fact that self-driving cars still operate in numerous stochastic environmental conditions including perception of the surrounding (object detection, identification, pedestrian and road cue detection etc), multiple road structures and routes, complicated mix of vehicles and interaction with other human-driven vehicle might makes them as complex and requires the consideration of numerous parameters. But during racing even though the speed is very high to achieve less lap time, the track does not have no passenger involved and the routes are pre-determined (Figure 1-Page 9/39).*

Our response:

We understand the confusion and the point of the reviewer and we updated our argument (lines 35-49) to reflect on the issue raised by the reviewer and included the arguments of the reviewer in the manuscript. Note that our argumentation was not intended to picture one problem more complex than the other but perhaps our message was not conveyed clearly. We hope in this revised version we have made it clear that we simply think they are optimised to drive in different conditions and environments which affects their experience and capabilities. For instance, self-driving passenger cars are not optimised to drive in a full-speed mode outside of highways (where the roads are very straight and not curvy) and therefore their models may not have the necessary experience to deal with such situations if for some unexpected reasons they find themselves in such situations.

2. *Lines (48-65) and (66-83) are repeated.*

Our response:

Thank you for pointing it out. It seems that when performing the final step of proof reading, this section was left duplicate. We are very sorry for letting this pass in the final version.

3. *What are the requirements to select TORCS as the main software candidate for simulation as it has many limitations with its Physics engine? (line 317)*

Our response:

Thanks for the suggestion. We also reworked the section Autonomous Racing and we described the advantages and disadvantages of using an open source simulator (TORCS) compared to a professional racing simulator to make it more clear.

4. *Participants (444): Based on the result of the simulation, the skilled (2 participants-455) achieve the EXCELLENT time lap as compared to the other 11 participants in a clearer manner. But during clustering (line-481) skill-based behavior was not selected as one to train the RL (Reinforcement learning). Why?*

Our response:

See response 6

5. *Lines (488-493) and lines (482-487) is repeated.*

Our response:

Thank you for pointing it out. It seems that when performing the final step of proof reading, this section was left duplicate. We are very sorry for letting this pass in the final version.

6. In the conclusion and in the body of the article (line 877-879 and lines 534-536), VERY GOOD performance driver's frustration even is higher as compared to MEDIUM and GOOD performance driver's from interview and was cross checked with TLX Mental demand. Not clear!

Our response:

- Calculating effect sizes, the effect of the Mental Demand, Performance, and Frustration comparisons are small, while the effect observed for Effort is large. The overall comparison is causing confusion regarding its utility for reinforcement learning. Therefore, we decided to retain the only conclusions regarding Effort. Participants in the EXCELLENT performance level reported lower perceived effort than those in the VERY GOOD and GOOD levels.
- Our assumption is that the driver makes a mental plan where the car has to be in order to achieve the best possible performance, but as the real execution differs the driver is trying to adjust while perceiving the errors. A driver performing at the best level perceives success in the formulated plan, which is translated in less correction effort. However, with the current evidence, this is merely an assumption and more in-depth study is needed to investigate cognitive effects. Connecting the TXL Effort result with the RL algorithm performance, Figure 14 shows the algorithm *effort* in terms of steps and laps needed at each performance level. In these terms, 1/3 of the training time is spent until the agent can drive the track at GOOD level, but it takes the algorithm 2/3 of the training time to progress from good to excellent. Thus, the comparable effort increases when trying to progress to the high performance levels. [we have changed the text in lines **843-859 and 513 - 520**]

7. In ensuring balanced grouping, the method employed (Kruskal wallis test) was unclear! And what is the result of computing TLX mental demand correlation's (Figure 3) effect even if the telemetry data of each participants does not match best with RL model?

Our response:

These are two questions: a) dealing with unbalanced data. b) drawing a relationship between subjective measure of mental demand and the RL.

a) Instead of using a parametric omnibus test (ANOVA), we used a Kruskal Wallis test followed by Dunn pairwise comparisons. This is due to the fact that the groups, in this case the performance categories, have different numbers of measurements (they are unbalanced). Kruskal-Wallis and Dunn pairwise comparisons are recommended tests for such cases by various textbooks. For

instance (Zar, Biostatistical Analysis, 5th, 2010). Notice that Dunn Test is comparably more conservative than, for example Tukey HSD.

We did not compute or report correlations. There was an error in the way the test statistics were reported, for example Dunn's Test revealed sig. differences ($p < .02, r = .56$). Dunn's Test uses a Z-statistic and the value reported corresponds to the z score. It should read ($p < .02, z = .56$). This has been corrected in the text.

b) Figure 3 is intended to illustrate the difference in perceived dimensions of workload when performing at each performance level. Figure 3 is difficult to read. It has been replaced by two figures. A general TLX summarizing scores across performance groups. A specific TLX per session and group.

8. *Line (574), breaking should be BRAKING*

Our response:

Thank you for pointing it out. We have fixed the typo.

9. *Line (745), we have two 'the'*

Our response:

Thank you for pointing it out. We have fixed the typo.

10. *Even though the Selected model learns to achieve the best time (98.91s -line 778), it still fails to predict any meaningful lap time (line 813 and line 825). If that is so, what is the strong reason to choose RL algorithm to choose to drive the autonomous driver? (850-853)*

Our response:

The question is referring to different models. The model that achieves the best lap-time is a reinforcement learning agent *(98.91s **-line 749**)* trained end-to-end with the simulator. This model can steer the car achieving the best lap time. The model used to predict laptime from telemetry *(line **784 and line 786**)* is a linear regression trained on the data obtained from humans. We tried to predict the laptime from the telemetry of the RL using the human-model, and this was unsuccessful. Therefore, it is necessary to train RL end-to-end. We use the racing line obtained by the best human driver to parametrize the reward function of the RL.

Furthermore, the failure of the human model to predict laps from RL is due to the fact that humans have different physical constraints in the response time compared to RL agents which can act much faster and can use controls more precisely. In the scenario where human drivers would try to learn from RL agents, as pointed in the lines **826-837**, then the actions of RL agents should be limited to reflect actions that can be taken from a human driver before training the RL model. We do not put such restrictions in place since we are not targeting such a use case, but in these situations we highly recommend self-racing developers to pose such restrictions as pointed in lines **826-837.**
